# Photoinduced large polaron transport and dynamics in organic–inorganic hybrid lead halide perovskite with terahertz probes

**DOI:** 10.1038/s41377-022-00872-y

**Published:** 2022-07-06

**Authors:** Zuanming Jin, Yan Peng, Yuqing Fang, Zhijiang Ye, Zhiyuan Fan, Zhilin Liu, Xichang Bao, Heng Gao, Wei Ren, Jing Wu, Guohong Ma, Qianli Chen, Chao Zhang, Alexey V. Balakin, Alexander P. Shkurinov, Yiming Zhu, Songlin Zhuang

**Affiliations:** 1 Terahertz Technology Innovation Research Institute, Terahertz Spectrum and Imaging Technology Cooperative Innovation Center, Shanghai Key Lab of Modern Optical System, University of Shanghai for Science and Technology, Shanghai, 200093 China; 2 Qingdao Institute of Bioenergy and Bioprocess Technology, Chinese Academy of Sciences, Qingdao, 266101 China; 3 Physics Department, Materials Genome Institute, State Key Laboratory of Advanced Special Steel, Shanghai Key Laboratory of High Temperature Superconductors, International Centre of Quantum and Molecular Structures, Shanghai University, Shanghai, 200444 China; 4 Shanghai Institute of Technical Physics, Chinese Academy of Sciences, Yutian Road 500, Shanghai, China; 5 Department of Physics, Shanghai University, 99 Shangda Road, Shanghai, 200444 China; 6 University of Michigan – Shanghai Jiao Tong University Joint Institute, Shanghai Jiao Tong University, Shanghai, China; 7 School of Physics, University of Wollongong, Wollongong, NSW 2522 Australia; 8 Department of Physics and International Laser Center, Lomonosov Moscow State University, Leninskie Gory 1, Moscow, 19991 Russia; 9 ILIT RAS-Branch of the FSRC《Crystallography and Photonics》RAS, Svyatoozerskaya 1, 140700, Shatura, Moscow Region, Russia

**Keywords:** Terahertz optics, Optical spectroscopy

## Abstract

Organic-inorganic hybrid metal halide perovskites (MHPs) have attracted tremendous attention for optoelectronic applications. The long photocarrier lifetime and moderate carrier mobility have been proposed as results of the large polaron formation in MHPs. However, it is challenging to measure the effective mass and carrier scattering parameters of the photogenerated large polarons in the ultrafast carrier recombination dynamics. Here, we show, in a one-step spectroscopic method, that the optical-pump and terahertz-electromagnetic probe (OPTP) technique allows us to access the nature of interplay of photoexcited unbound charge carriers and optical phonons in polycrystalline CH_3_NH_3_PbI_3_ (MAPbI_3_) of about 10 μm grain size. Firstly, we demonstrate a direct spectral evidence of the large polarons in polycrystalline MAPbI_3_. Using the Drude–Smith–Lorentz model along with the Frӧhlich-type electron-phonon (e-ph) coupling, we determine the effective mass and scattering parameters of photogenerated polaronic carriers. We discover that the resulting moderate polaronic carrier mobility is mainly influenced by the enhanced carrier scattering, rather than the polaron mass enhancement. While, the formation of large polarons in MAPbI_3_ polycrystalline grains results in a long charge carrier lifetime at room temperature. Our results provide crucial information about the photo-physics of MAPbI_3_ and are indispensable for optoelectronic device development with better performance.

## Abstract (web summary)

Ultrafast terahertz conductivity spectroscopy reveals the formation of large polarons in CH_3_NH_3_PbI_3_, which protects the charge carriers from scattering with polycrystalline grain boundaries or defects and explains the long lifetime.

## Graphical abstract

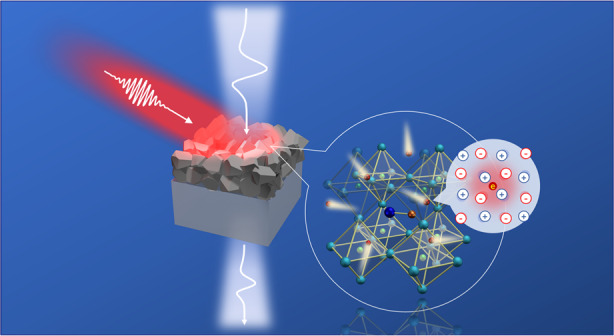

## Introduction

Organic–inorganic metal-halide perovskites (MHPs) are composed of organic cations and an inorganic framework. The success application of low-cost and solution-processable MHPs in optoelectronic devices can be attributed to low carrier recombination rates^1,2^, ambipolar carrier transport^3,4^, strong optical absorption^5,6^, long carrier diffusion^7–9^, and inherent defect tolerance^10^. Given these advantages over conventional semiconductors, MHPs have attracted tremendous scientific research interest in recent ten years^11,12^, for promising applications in cost-effective solar cells^13–16^, solid-state lighting^17–20^, transistors^21^, memristors^22^, and ultrafast spin switches in spintronics^23,24^.

Despite that MHPs behave as defect-free semiconductors with low charge recombination rates, long diffusion lengths (1–3 μm), and long hot carrier lifetimes (1–3 μs) that exceed the Langevin limit for direct recombination, these materials show a moderate free charge-carrier mobility (*μ* ∼ 30–100 cm^2^ V^−1^S^−1^) comparing to other inorganic semiconductors. Efforts have been made to figure out such an apparent discrepancy in the dynamical carrier properties in MHPs^25–27^. At room temperature, the interplay between carriers and ionic perovskite lattice is a possible microscopic mechanism behind this unusual recombination of photogenerated carriers in MAPbI_3_^28^. It was first proposed by Zhu and co-workers that large polarons—described as the charge carriers associated with the lattice deformation over several structural units—prevent scattering of photocarriers from charged defects, the remaining phonon bath, and other charge carriers in MHPs^29–32^.

A number of spectroscopic and optical measurements, such as time-resolved optical Kerr effect spectroscopy, time-resolved two-photon photoemission, transient reflectance, and absorption spectroscopies, time-domain Raman spectroscopy, angle-resolved photoelectron spectroscopy, have been used to reveal the electron-phonon (e-ph) coupling in CsPbBr_3_^32–34^, MAPbBr_3_^35–37^, and MAPbI_3_^38–42^. However, these measurements are associated with the large polaron formation either from the phonon or from the electronic structure perspective^42–44^ (See Supplementary Table S1 for a literature survey). In addition, it is challenging, to use an ultrafast spectroscopic technique in the visible region to access the polaron mobility, carrier scattering parameters, and dynamics in MHPs quantitatively, which are considered as the fundamental nature of the large polaron^29,45^.

In comparison with visible light, the frequencies of terahertz (THz) waves (ranging from several to tens of meV in energy scales) coincide with both the momentum relaxation times of conduction carriers^46,47^ and the typical optical phonons in MHPs^48–50^. Thus, ultrafast THz spectroscopy is expected to be an appropriate approach to study the low energy excitations and the coupling between charge carriers and phonons. However, until now, researchers have relied on two THz spectroscopic approaches to characterize the large polaron behaviors in MHPs. On the one hand, the rise of the THz photoconductivity dynamics is observed after hot-carrier cooling, which has been ascribed to an indirect evidence of polaron formation^51^. On the other hand, the temperature dependence of scattering time $\sim {{{T}}}^{ - 3/2}$ supports a strong dependence of carrier transport on the phonons^52–54^. This experimental behavior is inconsistent with the Fröhlich model, which predicts a temperature dependence $\sim {{{T}}}^{ - 0.46}$ of the polaron mobility^55^. Therefore, the full potential of THz spectroscopic measurements for direct evidence of polaron formation in MAPbI_3_ still remains to be shown.

We may also mention that quantitative characterization of the large polaron transport and dynamics in MHPs, requires the effective mass of the photoinduced charge carriers with renormalization factors due to polaron formation. A direct experimental method for determining the polaron mass in MHPs is the magneto-optical spectroscopy by observing the Landau levels^56,57^. For such measurements, a pulsed magnetic-field of 130 T^56^ and a low temperature down to 1.5 K^57^, are necessary. However, the small increase in the polaron mass cannot account for the moderate carrier mobility measured in MAPbI_3_^57^. Therefore, it is necessary to develop the capability to evaluate the backscattering probability of the carriers by traps and grain (or domain) boundaries on the ultrafast timescale^58,59^. Nevertheless, a one-step characterization satisfying the requirements of room-temperature, magnets-free, access to the basic large polaron transport and ultrafast dynamics quantitatively is expected to be important, but has not been shown.

In this letter, we present a direct spectroscopic proof for the large polaron behavior due to the dynamic coupling between optical phonons and photocarriers in prototypical MAPbI_3_ polycrystalline grains. To determine the large polaron mass and mobility, we take a completely different approach. We performed a contactless optical pump-THz electromagnetic probe (OPTP) measurement to obtain the complex-valued THz photoconductivity spectra ${{{\tilde{\mathrm \sigma }}}}_s^{\rm{pump}}\left( \omega \right)$ of MAPbI_3_. The resonance-like spectral line shapes of the photoexcited THz conductivity response are inherently indicative of the coupling between the optical phonon modes and the photoinduced carriers, considered as large polarons. Following the Drude–Smith–Lorentz (DSL) model and the Fröhlich-type large polaron model, we are able to extract the effective mass and the carrier scattering parameters of the large polaron. Consequently, the large polaron mobility of MAPbI_3_ is determined. It is found that the mass enhancement by polaron effect appears not to be significantly detrimental to the relatively low carrier mobility in MAPbI_3_. Taking advantage of the OPTP time-resolved spectroscopy, we further demonstrate that the long-lived large polaronic carriers are the major photogenerated species in MAPbI_3_ polycrystalline grains, which allows for efficient charge extraction. Our work provides important insights into the large polarons in MHPs, which is essential for developing energy and optoelectronic applications with better controlled charge-lattice interaction, charge-carrier mobilities, and transistor switching times.

## Results

The samples used in this work are MAPbI_3_ polycrystalline grains and thin films. Firstly, high-quality MAPbI_3_ single-crystals were synthesized according to the literature^60–62^. The photographs of the MAPbI_3_ single crystal and the corresponding X-ray diffraction (XRD) pattern are given in Figs. S1 and S2, respectively. The prominent peaks at 14.6° and 28° correspond to (110) and (220) planes of MAPbI_3_ single crystal. As very little THz signal could transmit through the thick single crystal, the MAPbI_3_ single crystal was ground into isotropic polycrystalline grains. The average size of the polycrystalline grains spread on a double-sided adhesive tape is about 10 ± 5 μm, which was determined by both optical microscopy and THz near-field optical microscope, as shown in Figs. S3 and S4. As control samples, the details of MAPbI_3_ perovskite thin films preparation are shown in Note 3 and Fig. S5 in the Supplemental Materials.

**Fig. 1:**
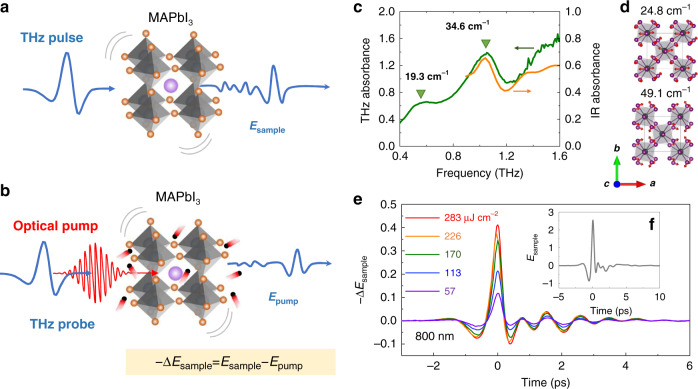
OPTP measurement scheme and sample characterizations. **a**, **b** Schematic illustration of the OPTP spectroscopy on MAPbI_3_. The quickly-oscillating pulse (red) is the optical pump beam, and the time-delayed single-cycle pulse (blue) represents the THz probe beam. **c** THz and FTIR absorbance spectra from 0.4 to 1.6 THz. **d** DFT calculated I-Pb-I bending motions at 24.8 and 49.1 cm^−^^1^ of MAPbI_3_. The gray and purple balls denote Pb and I atoms, respectively. **e** Time-domain signals transmitted through the MAPbI_3_ polycrystalline grains covered on a double-sided tape for different pump fluences (800 nm excitation). **f** A transmitted THz waveform through the sample without photoexcitation

We performed the ultrafast THz conductivity measurement using the OPTP technique, which probes the difference between pre-photoexcited and post-photoexcited THz conductivities of MAPbI_3_. As shown in Fig. 1a, b, an 800 nm (photon-energy of 1.55 eV) or a 400 nm (3.1 eV) laser beam was impinging on the MAPbI_3_ sample as an optical pump with a spot of ~5 mm in diameter. The photoexcited conductivity of the sample was probed with a sub-picosecond THz pulse. The transmitted THz probe pulse (containing spectral amplitude and phase information) through the sample was measured by an optical sampling pulse in a <110>-oriented ZnTe crystal of a thickness of 1 mm (see “Materials and methods”, Fig. S6).

The static THz properties of the unexcited perovskite sample were firstly investigated to elucidate the presence of transverse optical (TO) and longitudinal optical (LO) phonon modes (Fig. 1a), which is associated with the internal vibrations of the PbI_3_ framework^63^. Figure 1c shows the absorbance properties of the unexcited MAPbI_3_ polycrystalline grains, using both Fourier transform infrared spectroscopy (FTIR) and THz time-domain spectroscopy (THz-TDS) (see “Materials and methods” and Fig. S7 for details). Two vibrations in the THz absorbance spectrum around 0.58 THz (19.3 cm^−1^) and 1.04 THz (34.6 cm^−^^1^) originate mainly from the Pb–I inorganic sublattice. The THz absorption peak at 1.04 THz is associated with the IR-active mode. These two low-frequency skeletal bending modes can be assigned to the octahedral twist TO phonon mode and the octahedra distortion LO phonon mode, respectively^64^. We compare the experimental results with the DFT calculations, to elucidate the nature of these lattice vibration modes of MAPbI_3_ with tetragonal phase (*I4/mcm*). Figure 1d shows the calculated vibrational displacements for the 24.8 and 49.1 cm^−1^ modes of MAPbI_3_, which are composed of I–Pb–I bond bending motions mostly. They are assigned to B_1g_ and A_1g_ mode in the $D_{4h}^{18}$ symmetry group, respectively. The detailed calculations are shown in Fig. S9. Note that the DFT results show a ~20% deviation below 100 cm^−1 65^. The discrepancy between experimental data and theoretical model results can be attributed to the dynamic disorder of cations, cation rotational unlocking, and local polar fluctuation^66^.

To decipher the coupling of photoexcited carriers with the skeletal bending optical phonons^67,68^, the sample is excited by an 800 nm linear polarized pump laser pulse (Fig. 1b). Due to the photoexcitation, the transmitted THz waveform is modified. Figure 1e shows the pump-induced THz electric field change $- \Delta {{{E}}}_{{{{\mathrm{sample}}}}}$(*t*, Δ*t*), as a function of pump fluence at a given pump-probe time delay of Δ*t* = 5 ps. Figure 1f shows the time-domain THz waveform transmitted through the sample without photoexcitation, ${{{E}}}_{{{{\mathrm{sample}}}}}\left( t \right)$. The frequency-resolved complex sheet photoconductivity $\tilde \sigma _s^{{{{\mathrm{pump}}}}}({{{\mathrm{\omega }}}},\Delta {{{t}}}) = \tilde \sigma ^{{{{\mathrm{pump}}}}}\left( {{{{\mathrm{\omega }}}},\Delta {{{t}}}} \right) \times d_p$, where $d_p \approx 1\;\upmu{\rm{m}}$ is the penetration depth^69^. According to the applicability of the thin-film approximation in THz photoconductivity measurements^70^, $\frac{{n_2{{{\mathrm{d}}}}_p\omega }}{c} \approx 0.01 \ll 1$ for ω = 1 THz and *n*_2_ = 2.6 (*n*_2_ is the refractive index of non-photoexcited MAPbI_3_ at 300 K^71^) indicates that the thin film approximation holds. $\tilde \sigma _s^{{{{\mathrm{pump}}}}}({{{\mathrm{\omega }}}},\Delta {{{t}}})$ can be extracted by^72^,

1 $${{{\tilde{\mathrm \sigma }}}}_s^{{{{\mathrm{pump}}}}}\left( {\omega ,\Delta {{{t}}}} \right) = \frac{{n_1 + n_2}}{{Z_0}}\left( {\frac{1}{{T^{{{{\mathrm{pump}}}}}\left( {\omega ,\Delta {{{t}}}} \right)}} - 1} \right)$$

where, $T^{{{{\mathrm{pump}}}}}\left( {\omega ,\Delta {{{t}}}} \right) = \frac{{{{{E}}}_{{{{\mathrm{pump}}}}}\left( {\omega ,\Delta {{{\mathrm{t}}}}} \right)}}{{{{{\mathrm{E}}}}_{{{{\mathrm{sample}}}}}\left( \omega \right)}} = \frac{{{{{E}}}_{{{{\mathrm{sample}}}}}\left( \omega \right) + \Delta {{{E}}}_{{{{\mathrm{sample}}}}}\left( {\omega ,\Delta {{{t}}}} \right)}}{{{{{E}}}_{{{{\mathrm{sample}}}}}\left( \omega \right)}}$. *n*_1_ = 1 is the refractive index of air. *Z*_0_ = 377 Ω is the impedance of free space. *ω* is the THz angular frequencies of the probe electric fields. Note that the distinction between sheet and volume conductivity does not affect the calculation of the effective large polaron mass and mobility, which will be discussed later.

**Fig. 2:**
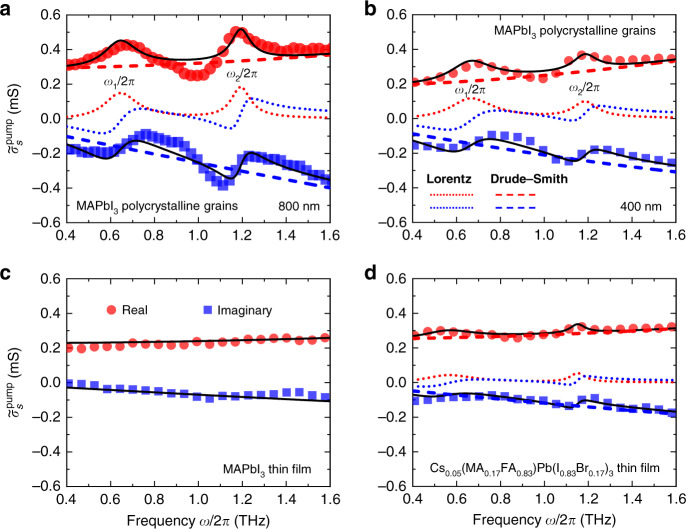
The observation of photoinduced large polaron in MAPbI_3_. Real (circles) and imaginary (squares) parts of the sheet photoconductivity spectra of MAPbI_3_ polycrystalline grains for two different pumped energies of **a** 1.55 eV and **b** 3.1 eV, at Δ*t* = 5 ps. The complex sheet photoconductivity spectra for **c** MAPbI_3_ thin film and **d** Cs_0.05_(MA_0.17_FA_0.83_)Pb(I_0.83_Br_0.17_)_3_ thin film with pumped energy of 3.1 eV, at Δ*t* = 5 ps. The black curves in (**a**), (**b**), and (**d**) represent the Drude–Smith–Lorentz fits. The dashed and dotted lines show the components for the Drude–Smith term and the Lorentz terms, respectively

Figure 2a, b shows the real part $\sigma _{{{{\mathrm{real}}}},{{{\mathrm{s}}}}}^{{{{\mathrm{pump}}}}}$(*ω*) and the imaginary part $\sigma _{{{{\mathrm{imag}}}},{{{\mathrm{s}}}}}^{{{{\mathrm{pump}}}}}$(*ω*) of the frequency-resolved ${{{\tilde{\mathrm \sigma }}}}_s^{{{{\mathrm{pump}}}}}$(*ω*, Δ*t* = 5 ps) of MAPbI_3_ polycrystalline grains, acquired at two pump wavelengths of 800 and 400 nm. The pump fluences are 57 μJ cm^−2^ (800 nm excitation) and 99 μJ ${{{\mathrm{cm}}}}^{ - 2}$ (400 nm excitation). Generally, the positive $\sigma _{{{{\mathrm{real}}}},{{{\mathrm{s}}}}}^{{{{\mathrm{pump}}}}}$(*ω*) grows with the increase in frequency, while the $\sigma _{{{{\mathrm{imag}}}},{{{\mathrm{s}}}}}^{{{{\mathrm{pump}}}}}$(*ω*) is negative and decreases with the increasing frequency, in the probed frequency window. The non-Drude spectral response clearly indicates that the free mobile charge is not the only contribution to the signal^73^. Because the thermal energy of 26 meV at room temperature is higher than the exciton binding energy of MAPbI_3_ (~10–16 meV)^74^, excitons are mostly dissociated into free carriers, and therefore any possibility of exciton contribution to the spectrum modulation can be excluded^75^. Since no external fields or electrodes are applied to the material during the measurement, the observed negative $\sigma _{{{{\mathrm{imag}}}},{{{\mathrm{s}}}}}^{{{{\mathrm{pump}}}}}$(*ω*) implies the presence of carrier scattering from the crystalline boundaries or defects, as characterized later^76^. It is important to note that, as shown in Fig. 2a, two resonant peaks in $\sigma _{{{{\mathrm{real}}}},{{{\mathrm{s}}}}}^{{{{\mathrm{pump}}}}}$(*ω*) accompanied by two inflection points in $\sigma _{{{{\mathrm{imag}}}},{{{\mathrm{s}}}}}^{{{{\mathrm{pump}}}}}$(*ω*) are observed at ~0.65 and ~1.17 THz, when excited with an 800 nm pump pulse. Similar resonant peaks are observed for the photoexcitation with a 400 nm pump pulse (Fig. 2b). This confirms the robustness of our observations and suggests that the photoconductivity spectrum is similar for band edge- (800 nm) and far-above-gap (400 nm) excitations, despite the difference in the excitation/relaxation channels. For comparison, the photoconductivity spectrum of a solution-processed MAPbI_3_ thin film (with grain size of several hundred nanometers) has been measured, as shown in Fig. 2c. No clear THz peaks on $\sigma _{{{{\mathrm{real}}}},{{{\mathrm{s}}}}}^{{{{\mathrm{pump}}}}}$(*ω*) are observed, consistent with the spectrum reported in similar MAPbI_3_ thin film^77^. This is due to the thermal or structural instabilities for the pure perovskite compound MAPbI_3_ thin film. The crystallization quality may be less good in the MAPbI_3_ thin film, leading to less strong optical phonon signal. A Drude–Smith (DS) model provides a quantitative description of the localized charge carriers in the MAPbI_3_ thin film (see Fig. S10). We further investigate a triple cation mixed halide (Cs)/MA/${{{\mathrm{CH}}}}_3({{{\mathrm{NH}}}}_2)_2^ +$(FA) thin film with better stability^78^. Figure 2d shows the presence of resonance-like modulations in $\sigma _{{{{\mathrm{real}}}},{{{\mathrm{s}}}}}^{{{{\mathrm{pump}}}}}$(*ω*) accompanied by inflection points in $\sigma _{{{{\mathrm{imag}}}},{{{\mathrm{s}}}}}^{{{{\mathrm{pump}}}}}$(*ω*) of the Cs_0.05_(MA_0.17_FA_0.83_)Pb(I_0.83_Br_0.17_)_3_ thin film (see Fig. S11 for details). The observed feature of resonances in the photoconductivity spectra comes from the coupling the photoexcited charge carriers with the optical phonons of the soft lattice^79,80^.

To gain more insight into our data, ${{{\tilde{\mathrm \sigma }}}}_s^{{{{\mathrm{pump}}}}}\left( \omega \right)$ is fitted by a DSL model,

2 $${{{\tilde{\mathrm \sigma }}}}_s^{{{{\mathrm{pump}}}}}\left( \omega \right) = \frac{{{{{\mathrm{\varepsilon }}}}_0\omega _{{{\mathrm{p}}}}^2}}{{{{\Gamma }} - {{{\mathrm{i}}}}\omega }}\left( {1 + \frac{{{{{\mathrm{c}}}}_1}}{{1 - {{{\mathrm{i}}}}\omega /{{\Gamma }}}}} \right) + \mathop {\sum }\limits_{{{\mathrm{m}}}} \frac{{{{{\mathrm{g}}}}_{{{\mathrm{m}}}}\omega }}{{{{{\mathrm{i}}}}\left( {\omega _{{{\mathrm{m}}}}^2 - \omega ^2} \right) + {{{\mathrm{\gamma }}}}_{{{\mathrm{m}}}}\omega }}$$

The first term of Eq. (2) indicates the DS portion, which accounts for the disorder or backscattering induced transport of mobile charge carriers^81–83^. *ε*_0_ is the vacuum permittivity. The DS term has three free parameters *ω*_p_, c_1_ and Γ. *ω*_p_ is the Drude plasma frequency. *c*_1_ is the phenomenological localization parameter, which ranges between 0 and −1. 0 and −1 manifest free-carrier transport and 100% backscattering, respectively. Γ denotes the DS scattering rate of the free carriers, related to the carrier mobility by $\mu = e\left( {1 + c_1} \right)/(\Gamma m_p^ \ast )$, where $m_p^ \ast$ is the effective mass of polarons in MHPs and *e* is the elementary charge. The second term of Eq. (2) is the Lorentzian portion. *g*_m_, *γ*_m_, and *ω*_m_ are the oscillator strength, full-width at half-maximum (FWHM) linewidth, and THz angular frequency of the optical phonon modes m, respectively. Note that the global fitting of DSL model is performed on both measured $\sigma _{{{{\mathrm{real}}}},{{{\mathrm{s}}}}}^{{{{\mathrm{pump}}}}}$(*ω*) and $\sigma _{{{{\mathrm{imag}}}},{{{\mathrm{s}}}}}^{{{{\mathrm{pump}}}}}$(*ω*) simultaneously, over the entire measured spectra.

To aid comparison, we separate the DS component (dashed lines) and Lorentz component (dotted lines) of the ${{{\tilde{\mathrm \sigma }}}}_s^{{{{\mathrm{pump}}}}}\left( \omega \right)$ (Fig. 2a, b). The extracted resonance frequencies of the optical phonon modes, $\omega _1/2\pi \approx\,21.6\, {{\rm{cm}}^{-1}}$, $\omega _2/2\pi \approx\, 39.0\, {\rm{cm}^{-1}}$ (800 nm pump), and $\omega _1/2\pi \approx\,22.3\,{{\rm{cm}}^{-1}}$, $\omega _2/2\pi \approx\,39.3\,{{\rm{cm}}^{-1}}$ (400 nm pump). Both are blue-shifted compared to the static optical phonon modes of 19.3 and 34.6 cm^−^^1^ (Fig. 1c). It is noteworthy that the resonance peaks around 21.6 and 39.0 cm^−^^1^ probed by OPTP spectroscopy are in good agreement with the excited-state wave packets oscillating at ~21 and ~45 cm^−^^1^ measured by time-domain Raman spectroscopy^39^, as well as the strong e-ph coupling at 21.6 and 40 cm^−^^1^ obtained by THz emission spectrum^84^.

**Fig. 3:**
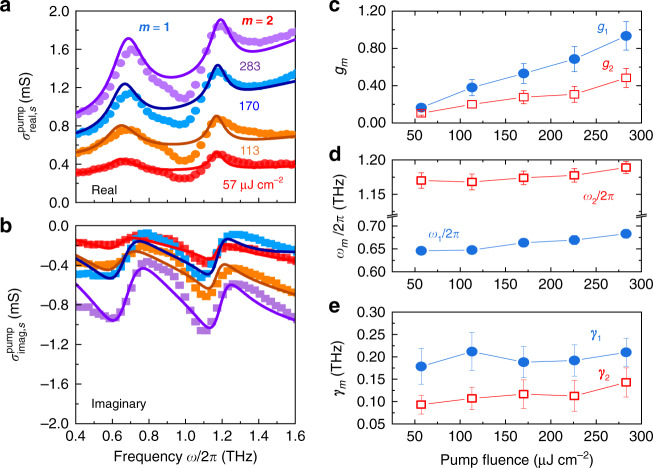
Pump fluence-dependent THz photoconductivity spectra for MAPbI_3_ polycrystalline grains. Extracted **a** real and **b** imaginary parts of THz photoconductivity spectra of MAPbI_3_ polycrystalline grains, following photoexcitation at 1.55 eV with pump fluences of 57, 113, 170, and 283 μJ cm^−2^ at 300 K. The solid lines are the DSL model fits. Pump fluence evolutions of **c** oscillator strength *g*_m_, **d** resonant frequency $\omega _{{{\mathrm{m}}}}/2\pi$, and **e** FWHM linewidth *γ*_m_ of the resonance modes

To further reveal the nature of the e-ph interaction, we analyze the ${{{\tilde{\mathrm \sigma }}}}_s^{{{{\mathrm{pump}}}}}\left( \omega \right)$ using the 800 nm excitation at Δ*t* = 5 ps with different pump fluences, as shown in Fig. 3a, b. Our experimental results can be well fitted by the DSL model (see Fig. S12 for details of the DS and Lorentz components). Remarkably, the increase of pump fluence modifies the spectral line-shape and amplitude of the resonances. Figure 3c–e summarizes the extracted oscillator strength (*g*_1,2_), mode frequency ($\omega _{1,2}/2{{{\mathrm{\pi }}}}$), and FWHM linewidth ($\gamma _{1,2}$) of two photo-induced optical phonon modes versus the pump fluence. It can be identified that *γ*_1_ is broader than *γ*_2_, while both are independent of pump fluence within the given uncertainty. *g*_1,2_ increases upon increasing the pump fluence. In addition, $\omega _1/2{{{\mathrm{\pi }}}}$ and $\omega _2/2{{{\mathrm{\pi }}}}$ are slightly blue-shifted from 0.65 ± 0.017 to 0.69 ± 0.003 THz and from 1.17 ± 0.019 to 1.19 ± 0.004 THz, respectively, when the pump fluence increases from 57 to 283 μJ cm^−^^2^. According to the DFT simulations, the neutral geometry of perovskite MAPbI_3_ is nearly a regular octahedron. Once a charge is introduced into the lattice, it results in a more ionic Pb–I framework and structural distortion, leading to a blueshift of phonon modes^80^. These observations corroborate again our claim that a direct fingerprint of a polaron formation is demonstrated, and will be used below to obtain the effective mass and the mobility of large polaron.

For now, let us concentrate on the effective mass enhancement (compared with the bare band effective mass) of the large polaron in MAPbI_3_, due to e–ph interaction at zero magnetic field, first proposed by Feynman^85–87^

3 $$m_p^ \ast = m_b^ \ast \left(1 + \frac{{\alpha _{{\rm{e - ph}}}}}{6} + \frac{{\alpha _{{\rm{e - ph}}}^2}}{{40}} + \ldots \right)$$

where $m_b^ \ast = 0.1 \times m_0$ (*m*_0_: the mass of free electron) is the bare band effective mass of electrons in solids, $\alpha _{\rm{{e - ph}}}$ is the dimensionless Fröhlich e–ph coupling constant, given by the Landau–Pekar model^43,85,86,88^:

4 $$\alpha _{\rm{{e - ph}}} = \frac{{e^2}}{{4\pi \hbar }}\left( {\frac{1}{{\varepsilon _{\rm{opt}}}} - \frac{1}{{\varepsilon _s}}} \right)\sqrt {\frac{{m_b^ \ast }}{{2\hbar \omega _{\rm{ph}}}}}$$

here, $\varepsilon _{\rm{opt}} \approx 5.5\varepsilon _0$ and $\varepsilon _s \approx 30\varepsilon _0$ are the optical and static dielectric constants^67^. $\left( {\frac{1}{{\varepsilon _{\rm{opt}}}} - \frac{1}{{\varepsilon _s}}} \right)$ qualitatively manifests the capacity of the screening due to a Coulomb potential of the polarized lattice. $\omega _{\rm{ph}}$ is the angular frequency of the LO phonon mode and $\hbar$ is the reduced Planck constant. By using the LO phonon mode of $34.6\;{{{\mathrm{cm}}}}^{ - 1}$ from our measurement, we obtain $\alpha _{\rm{{e - ph}}}\approx 2.64$ in MAPbI_3_ polycrystalline grains. This number is significantly larger than 0.07 for GaAs and 0.29 for CdTe, and is smaller than 6, which indicates the formation of large polaron in MAPbI_3_ materials^55^. The effective large polaron mass is consequently calculated, $m_p^ \ast=0.144\,m_0$. To proceed, using the mode frequencies of photoinduced LO phonon resonance ($\omega _{\rm{ph}}/2\pi \approx 39.0-39.6\;{{{\mathrm{cm}}}}^{ - 1}$) with increased pump fluences, $\alpha _{\rm{{e - ph}}}$ decreases by about 6.6% from about 2.64 to 2.46. Meanwhile, $m_p^ \ast$ decreases by about 2% from about 0.144 *m*_0_ to 0.141 *m*_0_. These numbers fall well within the range of the large polaron mass determined by both magneto-optical spectroscopy^56,57^ and calculations^89,90^. We would comment that the mass enhancement by polaron effect decreases the mobility by about 30.6%, which cannot exclusively account for the moderate carrier mobility of MAPbI_3_. Following the DSL model Eq. (2), the mean carrier scattering rate $\Gamma \approx 25.8 \pm 6.0\;{{{\mathrm{THz}}}}$ and the localization factor (1 + *c*_1_) $= 0.17 \pm 0.04$ have been extracted (see Fig. S12). The relation $\mu = e\left( {1 + c_1} \right)/(\Gamma m_p^ \ast )$ allows one to calculate the large polaron mobility on the order of ~80.7 ± 26.0 cm^2^ V^−1^s^−1^, which reaches up to ~60.7% of the theoretical limit^55^. The localization factor (1 + *c*_1_) due to the back-scattering by grain boundaries or defects further suppresses the mobility by ~83%. The mobility value is comparable with the values of MAPbI_3_ single crystal and polycrystalline films, measured by both THz and microwave spectroscopy, as summarized in Table 1^91–96^. We note that the $m_p^ \ast$ used here is not assumed a priori but rather determined experimentally. In our case, both the mass enhancement of large polaron and the boundary or defect scattering account for the relatively low polaron-based mobility in MAPbI_3_ polycrystalline grains. It is expected that the polaronic carriers can transport more efficiently with less structural disorder in MAPbI_3_.

**Table 1:** Comparison of the Fröhlich coupling constant $\alpha _{\rm{{e - ph}}}$, polaron effective mass $m_p^ \ast$ (relative to the band effective mass $m_b^ \ast$) and mobility measured in MAPbI_3_ with that from calculations and measurements in the literatures

Material	${{{\mathrm{\alpha }}}}_{{{{\mathrm{e}}}} - {{{\mathrm{ph}}}}}$	$m_p^ \ast$ (%)	Mobility at room temperature (cm^2^ V^−^^1^ s^−^^1^)	Approach	Theoretical or experimental
MAPbI_3_ polycrystalline grains (this work)	~2.6–2.46	+41–44%	~80.7 ± 26.0	Time-resolved THz spectroscopy	Experimental
MAPbI_3_	2.39	+37%	136	Single-phonon Fröhlich model	Theoretical^55^
MAPbI_3_	1.4	+28%		Multiphonon Fröhlich polaron model	Theoretical^93^
MAPbl_3_	1.91		33	Multi-LO phonon coupling	Theoretical^94^
MAPbI_3_ thin film	1.72	+35%	197	Far-IR spectroscopy	Experimental^85^
MAPbI_3_ single crystal	2			Time-resolved multi-THz spectroscopy	Experimental^67^
MAPbI_3_ thin film			20–75	Time-resolved microwave conductivity	Experimental^95^
MAPbI_3_ thin film			~25	Time-resolved THz spectroscopy; Time-resolved microwave conductivity	Experimental^46^
MAPbI_3_ thin film			~27	Time-resolved THz spectroscopy	Experimental^53^
MAPbI_3_ single crystals			59	Time-resolved THz spectroscopy	Experimental^96^
MAPbI_3_ thin film			33		

$m_p^ \ast$ (%): in terms of its increase with respect to the band effective mass

**Fig. 4:**
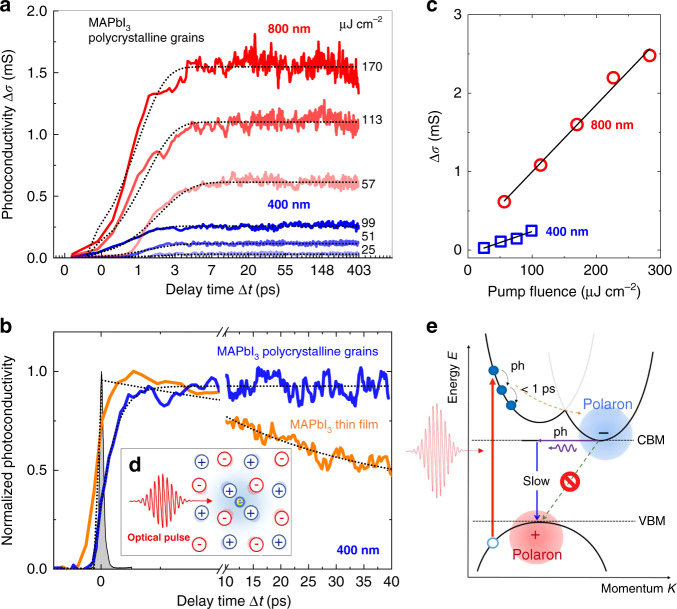
Ultrafast large polaron formation and relaxation dynamics of MAPbI_3_. **a** The transient THz photoconductivity of the MAPbI_3_ polycrystalline grains measured at 800 nm (red) and 400 nm (blue) laser excitations, as plotted in the log scale. **b** Time-resolved THz photoconductivity dynamics normalized to 1, for two different samples, using excitation of 400 nm wavelength. The gray profile represents the pump pulse. **c** The pump fluence dependences of the quasi-steady-state THz photoconductivity. The solid line is a fit to the data linearly. **d** A large polaron in MAPbI_3_ looks like a lump of negative charge that attracts MA^+^ cations and repels halide anions. **e** A proposed band diagram and photoexcitation dynamics in MAPbI_3_

We now move one step further in our analysis to explore the dynamics of photo-induced large polaron. Figure 4a shows the dynamics of the frequency-averaged photoconductivity Δσ(Δ*t*), measured with different pump fluences of 800 and 400 nm excitations (see “Materials and methods”). As shown in Fig. 4b, compared with the optical pump pulse, the rise of Δσ(Δ*t*) observed in MAPbI_3_ polycrystalline grains is slower than the timescale of the instrument-response function (gray). We note that Δσ(Δ*t*) increases in the first 5 ps due to the formation of conductive polaronic carriers until a quasi-steady-state is reached, which happens on a different timescale from the generation of free carriers that occurs on sub-100 fs timescales^97–99^. The slow rise of THz photoconductivity has been attributed to the charge carrier cooling and polaron formation sequentially^51,73^. A time-dependent polaron population model in ref. ^51^. is used to fit the rising dynamics of the polycrystalline sample with different pump fluences, as shown the dot lines in Fig. 4a. The time constants for the large polaron formation and the hot electron cooling are ~460 and ~180 fs, respectively, in good agreement with the values reported for similar MAPbI_3_ samples at room temperature^51^. Fig. 4c shows the linear dependence of the quasi-steady-state photoconductivity on the pump fluences in the range 25–283 μJ cm^−2^, which confirms the investigation of the large polaron in the linear regime. There was no observable photo-degradation and/or thermal damage in the polycrystalline MAPbI_3_ sample as the pump fluence reaches up to 1 mJ cm^−2^. However, there was no direct experimental characterization to clarify it, which will be our future research focus. Importantly, we note that ${{{\tilde{\mathrm \sigma }}}}_s^{{{{\mathrm{pump}}}}}\left( \omega \right)$ measured at ∆*t* = 200 ps shows a similar spectral characteristic as that measured at 5 ps just after photoexcitation (see Fig. S13). In addition, Δσ(Δ*t*) shows very stable and non-decaying dynamics within 400 ps (limited by current temporal probe window), indicating a very long-lifetime recombination process of large polaron-based carriers in MAPbI_3_ polycrystalline grains. In contrast, as the less significant polaronic effect observed in our solution-processed MAPbI_3_ thin film, the photoconductivity shows a faster decay and a much shorter lifetime (orange line in Fig. 4b). This behavior is likely caused by the charge trapping due to the defects and imperfections. We conclude that the formation of large polaron in MAPbI_3_ polycrystalline grains efficiently protects the charge carriers from being trapped, thus extends the carrier lifetime.

## Discussion

First, based on the experimental results, we directly identify the photocarriers-optical phonon coupling in different types of MAPbI_3_ samples by OPTP spectroscopy, revealing the underlying large polaron formation. Considering the DSL model and Frӧhlich-type large polaron, we demonstrate a straightforward experimental measurement of the effective mass, e–ph coupling constant, which are not presupposed. Using the extracted carrier scattering parameters, we derive the large polaron mobility of MAPbI_3_ polycrystalline grains. The moderate polaronic carrier mobility is mainly determined by the enhanced carrier scattering, rather than the polaron mass enhancement. In addition to the partial contribution to the limitation of the intra-grain charge transport, the presence of large polarons leads to the long charge carrier lifetime in MAPbI_3_. Our spectroscopic results of the large polaron formation rationalizes the previously reported contradiction between the long carrier lifetimes (slow electron–hole recombination) and the moderate carrier mobility in MAPbI_3_.

Second, our understanding on transport mechanism involving in both polaronic carrier mobility and lifetime in MAPbI_3_ provides a unifying picture of photo-excitation and relaxation channels in MAPbI_3_ qualitatively. Initially, the photons are absorbed in MAPbI_3_ to generate transient excitons, which can be effectively dissociated into free carriers^100^. In the real space, owing to the Coulomb interaction, a photo-induced carrier shifts the equilibrium positions of the surrounding anions and cations from their carrier-free positions^101^. The displacement between ions in the lattice results in a potential well, which traps the photocarriers, as illustrated in Fig. 4d. Within the energy-momentum space (Fig. 4e), the absorbed photons create a Fermi–Dirac distribution, which is thermalized by the carrier–carrier scattering on the 100 fs timescale^102,103^. The hot-carrier cooling is described as the electrons cascading down to the conduction band minimum, or the holes cascading up to the valence band maximum, exchanging ~meV energy with the lattice by LO phonon emission, on the timescale of 100 fs to 1 ps^104^. Meanwhile, the large polaron formation occurs at around 460 fs. The enhanced screening weakens the large polaron scattering with other carriers, defects, and LO phonons. Because of the inhibited LO-phonon emission, hot electron energy is retained and hot polarons are formed. The slow recombination of hot polarons that occurs on hundreds of picoseconds can be explained by the low thermal conductivity of MAPbI_3_^105,106^. As the length of charge diffusion is determined by the carrier recombination lifetime, the charge extraction is still suitable, despite the polaron mobility is relatively low. Thus, insights into the large polarons in the nonequilibrium higher energy states are crucial for developing efficient hot carrier-based optoelectronic applications^107^. As such, with the advance in device fabrication and characterization of MHPs using femtosecond laser techniques, the photodegradation and transient laser heating effect need to be addressed and will be the topic of future work.

Finally, we would comment that the experimental results are discussed in the framework of the Fröhlich polaron, which is based on the harmonic approximation. While, due to the dynamic disordered properties of the MAPbI_3_ lattice, the local electric field can produce local ordering of polar nanodomains surrounding the photocarriers^108^. Therefore, from the theoretical perspective, a more accurate model with local ferroelectric-like large polarons (including anharmonic and dynamical disorder effects)^109^ is desired to microscopically visualize the dynamics of large polarons. Furthermore, experimental efforts of optical pump-THz near field microscopy are required to characterize the transport of large polarons affected by defects and grain boundaries^110–112^.

In summary, the THz photoconductivity spectra of MAPbI_3_ polycrystalline grains have been modeled by the DS model augmented by Lorentzian terms, accounting for the fingerprint of optical phonon modes coupled to the photogenerated carriers. From our analysis that combines DSL model and Frӧhlich-type large polaron, no prior knowledges about the effective mass and scattering events are required to experimentally derive the large polaron mobility of MAPbI_3_. In addition, the formation of large polarons results in much longer charge carrier lifetime in polycrystalline MAPbI_3_, which is attributed to the efficient protection of band-edge carriers by self-trapping potential and low thermal conductivity. Our work adds to the fundamental understanding on the polaronic nature of charge carriers in MAPbI_3_ at room temperature, which is essential to their optoelectronic properties and applications.

## Materials and methods

### Samples preparation

To synthesize CH_3_NH_3_I (MAI), 24 mL of methylamine (33 wt% dissolved in EtOH), 10 mL of hydroiodic acid (57 wt% dissolved in H_2_O), and 100 mL of EtOH were added into a 250 mL-round bottom flask. The reaction took place under Ar gas at 0 °C for 2 h upon stirring. After reaction, a white product MAI was collected by rotary evaporation in a water-bath of 50 °C. Thereafter, the MAI precipitate was dissolved in EtOH and then underwent sedimentation in diethyl ether upon stirring for 30 min. After that, single crystals of MAPbI_3_ were prepared by first mixing PbI_2_ and MAI solution homogenously at a high temperature and then slowly cooling it down to an ambient temperature. Since the THz pulse can hardly transmit through a thick crystal, the MAPbI_3_ single crystal was ground into polycrystalline grains for the following investigation.

### Terahertz (THz) time-domain spectroscopy (THz-TDS)

Broadband THz-TDS was carried out on the MAPbI_3_ polycrystalline grains with the EKSPLA system. Femtosecond laser pulse (150 mW, 100 fs duration, 800 nm wavelength, 76 MHz repetition rate) was split into two beams: generation and detection laser beam. The generation beam was focused on a low temperature grown (LTG) GaAs photoconductive emitter. The generation beam was modulated by an optical chopper. The generated THz beam is focused by paraboloidal mirrors and transmitted through the MAPbI_3_ polycrystalline grains. The detection laser beam through a delay stage was used to record the THz waveform by another LTG GaAs photoconductive detector. The signal to noise ratio is better than 1000:1. All the static THz spectra were performed in the drying air environment at room temperature. The refractive index and absorption coefficient of the double-sided adhesive tape are shown in Fig. S8.

### Optical pump/THz electromagnetic probe (OPTP) spectroscopy

The OPTP system is driven by a Ti:sapphire fs amplifier laser pulse of ~120 fs duration with a repetition rate of 1 kHz. The laser beam was split into three arms: a pump beam, a THz probe beam, and an electro-optic sampling (EOS) beam (see Fig. S6). An 800 nm or 400 nm pump beam was used to photoexcite the MAPbI_3_ sample within a ~5 mm diameter area, ensuring a homogeneous pumping condition. The 400 nm pump beam is produced by second harmonic generation based on the 800 nm beam by beta barium borate (BBO) crystal. The time-resolved photo-induced complex THz conductivity spectra were interrogated using a time-delayed freely propagating THz probe transient with a ~1 ps-long optical cycle. The THz probe pulse was generated by an optical rectification crystal ZnTe (<110> orientation, 10 mm × 10 mm × 1 mm thickness), which was pumped with a slightly focused 800 nm laser beam. The THz beam was focused on the sample (~3 mm in diameter) using a pair of off-axis parabolic mirrors. The THz electric field accelerates the photocarriers, leading to an attenuation of the THz electric field. The transmitted THz pulses through the sample were recollimated and focused onto a second 1 mm-thick <110> ZnTe by another pair of parabolic mirrors. The modulated THz electric fields were coherently recorded by the free-space EOS beam, using a Wollaston prism, a pair of balanced photodiodes and a lock-in amplifier. Our OPTP setup is free of any spot-size effect and it is a homogeneous excitation for all frequencies in the THz pulse bandwidth. A custom-made drying air-flow chamber was used to prevent humidity-degradation of the MAPbI_3_ samples during the measurement as much as possible. No obvious observation of photodegradation and/or thermal damage was found during a 12 h experiment period. However, there was no further experimental characterization to clarify it. We also note that no photo-induced change can be observed in the THz transmission for a single double-sided tape. All measurements were performed at room temperature.

### Theoretical calculations of vibrational frequencies

We carried out the structural optimization and phonon calculations of MAPbI_3_ using the first principles within the framework of density functional theory (DFT). We used the DFT software of Vienna ab initio simulation package based on the projector augmented wave method. We treated the exchange-correlation interaction in the DFT calculations using the generalized gradient approximation which is proposed by Perdew, Burke, and Ernzerhof (PBE). We adopted a $\sqrt 2 \times \sqrt 2 \times 2$ MAPbI_3_ unit cell for structural optimization and phonon calculations. During the DFT simulations, we used the energy cutoff of 500 eV and a Monkhorst–Pack grid with 4×4×4 k-points. We set the convergence criterion of Hellman–Feynman force as 0.01 eV ${{{\mathrm{{\AA}}}}}^{ - 1}$ during the structural optimization. We used the density functional perturbation theory (DFPT) method to calculate the phonon frequencies and eigenvectors for Γ point of MAPbI_3_. The phonon vibrational displacements of the vibrational modes 24.8, 49.1, and 82.9 cm^−1^ are listed in Fig. S9 and Table S2.

## Supplementary information

Supplementary Information for Photoinduced large polaron transport and dynamics in organic-inorganic hybrid lead halide perovskite with terahertz probes

## Data Availability

The data that support the findings of this study are available from the corresponding author upon reasonable request.
