# Supplementary material for: Photoinduced large polaron transport and dynamics in organic–inorganic hybrid lead halide perovskite with terahertz probes
Source: Light Sci Appl. 2022 Jul 6;11:209. doi: 10.1038/s41377-022-00872-y (PMC9259629; doi:10.1038/s41377-022-00872-y)
Supplement: Supplementary file 1 — Supplementary Information for Photoinduced large polaron transport and dynamics in organic-inorganic hybrid lead halide perovskite with terahertz probes [file 41377_2022_872_MOESM1_ESM.docx]

**Supplementary Information for**

**Photoinduced large polaron transport and dynamics in organic-inorganic hybrid lead halide perovskite with terahertz probes**

Zuanming Jin^1^, Yan Peng^1,*^, Yuqing Fang^1^, Zhijiang Ye^1^, Zhiyuan Fan^1^, Zhilin Liu^2^, Xichang Bao^2^, Heng Gao^3^, Wei Ren^3^, Jing Wu^4^, Guohong Ma^5^, Qianli Chen^6^, Chao Zhang^7^, Alexey V. Balakin^8,9^, Alexander P. Shkurinov^8,9^, Yiming Zhu^1,*^, Songlin Zhuang^1^

*^1^Terahertz Technology Innovation Research Institute, Terahertz Spectrum and Imaging Technology Cooperative Innovation Center, Shanghai Key Lab of Modern Optical System, University of Shanghai for Science and Technology, Shanghai 200093, China*

*^2^Qingdao Institute of Bioenergy and Bioprocess Technology, Chinese Academy of Sciences, Qingdao 266101, China*

*^3^Physics Department, Materials Genome Institute, State Key Laboratory of Advanced Special Steel, Shanghai Key Laboratory of High Temperature Superconductors, International Centre of Quantum and Molecular Structures, Shanghai University, Shanghai 200444, China*

*^4^Shanghai Institute of Technical Physics, Chinese Academy of Sciences, Yutian Road 500, Shanghai, China*

*^5^Department of Physics, Shanghai University, 99 Shangda Road, Shanghai 200444, China*

*^6^ University of Michigan – Shanghai Jiao Tong University Joint Institute, Shanghai Jiao Tong University, China*

*^7^School of Physics, University of Wollongong, Wollongong, New South Wales 2522, Australia*

*^8^Department of Physics and International Laser Center, Lomonosov Moscow State University, Leninskie Gory 1, Moscow 19991, Russia*

*^9^ILIT RAS-Branch of the FSRC《Crystallography and Photonics》RAS, Svyatoozerskaya 1, 140700, Shatura, Moscow Region, Russia*

*Corresponding authors: py@usst.edu.cn, ymzhu@usst.edu.cn

**Table of Contents**

1. **Note 1.** Literature overview of the large polaron formation.
2. **Note 2.** MAPbI_3_ polycrystalline grains preparation and characterization.
3. **Note 3.** MAPbI_3_ thin film fabrication and characterization.
4. **Note 4.** Optical pump/THz probe (OPTP) spectroscopy.
5. **Note 5.** Terahertz time-domain spectroscopy (TDS).
6. **Note 6.** Theoretical calculations of vibrational frequencies.
7. **Note 7.** THz photoconductivity spectra of MAPbI_3_ thin film and Cs_0.05_(MA_0.17_FA_0.83_)Pb(I_0.83_Br_0.17_)_3_ thin film.
8. **Note 8.** THz photoconductivity spectra fitted by the DSL model.
9. **Note 9.** THz Photoconductivity of MAPbI_3_ polycrystalline grains measured at 5 ps and 200 ps.

**Note 1. Literature overview of the large polaron formation.**

|  | **Experimental measurements** | **Materials** | **Research contents** | **Polaron dynamics** | **Polaron transport** | **phonon / carrier perspective** | **References** |
| --- | --- | --- | --- | --- | --- | --- | --- |
| (1) | Time-resolved optical Kerr effect spectroscopy | single-crystal  CH_3_NH_3_PbBr_3_ and CsPbBr_3_ | The structural dynamics triggered by photo-carrier injection | yes | no | phonon | [32]  [35] |
| (2) | Ultrafast transient absorption  or reflection spectroscopy | CH_3_NH_3_PbBr_3_ and CsPbBr_3_;  FAPbI_3_ and Cs_0.01_FA_0.99_Pb(Br_0.11_I_0.89_)_3_ | The photoinduced absorption and bleach at specific energies  photo-induced bandgap renormalizaiton | yes | no | electronic structure | [32],  [36-38],  [44] |
| (3) | Time-resolved two-photon photoemission | single-crystal  CsPbBr_3_ and CsPbBr_3_ | The dynamic changes in the photoemission cross-section and in the red-shift of the optical bandgap | yes | no | carrier | [34] |
| (4) | Time-domain Raman spectroscopy | CH_3_NH_3_PbI_3_ | Examining Pb-I structural dynamics by oscillatory features in time-domain | yes | no | phonon | [39] |
| (5) | Angle-resolved photoelectron spectroscopy | CsPbBr_3_ single crystals | Signatures of large polaron formation in the electronic structure | no | no | electronic structure | [33] |
| (6) | Time-averaged  Photoluminescence spectroscopy | CH_3_NH_3_PbI_3_  and CH_3_NH_3_PbBr_3_ films | The temperature-dependent PL linewidth | no | no | phonon | [27] |
| (7) | Time-resolved photoluminescence spectra | HC(NH_2_)_2_PbI_3_  and CH_3_NH_3_PbI_3_ | The long lifetime of band-edge charge carriers | yes | no | carrier | [35]  [41] |
| (8) | Hall effect and  photoconductivity measurements | CH_3_NH_3_PbBr_3_ single crystals; CH_3_NH_3_PbI_3_ films | The density of photogenerated carriers and the intrinsic carrier mobility | no | yes | carrier | [30] |
| (9) | Ultrafast Terahertz (THz) spectroscopy | CH_3_NH_3_PbI_3_ thin films | The rise of the THz photoconductivity dynamics; Temperature dependence of scattering time | yes | yes | both carrier and phonon | [51-54] |

**Table S1. Comparison of the spectroscopic and optical measurements of the large polaron formation in the literatures.**

**Note 2.** **MAPbI_3_ polycrystalline grains preparation** **and** **characterization.**

CH_3_NH_3_I (MAI) powder was synthesized by reacting 24 mL of methylamine (33 wt% in ethanol) and 10 mL of hydroiodic acid (57 wt% in water, Aladdin reagent, Shanghai, China), and 100 mL ethanol in a 250 mL round bottom flask under argon at 0 $℃$ for 2 h with stirring. After the reaction, the white precipitate of MAI was recovered by rotary evaporation at 50 $℃$ and then dissolved in ethanol followed by sedimentation in diethyl ether by stirring the solution for 30 min. After that, MAPbI_3_ single crystals were grown via the inverse temperature crystallization method. Briefly, by mixing the PbI_2_ solution and MAI solution at a high temperature to get a homogenous solution, and by slowly cooling it down to room temperature, black MAPbI_3_ single crystals with large mirror-like facets can be attained. As very little THz signal could transmit through the thick crystal, the MAPbI_3_ single crystal was ground into polycrystalline grains.

**Figure S1** (a) show the image of the sample, which is a single crystal MAPbI_3_ approximately 2 ⨯ 2 mm in size.

**Figure S1**. Photograph of the (a) MAPbI_3_ single crystal and (b) MAPbI_3_ polycrystalline grains spread on the double-sided adhesive tape.

**Figure S2**. XRD measurement of MAPbI_3_ single crystal.

**Figure S2** shows the X-ray diffraction (XRD) pattern of MAPbI_3_ single crystal. The (110) and (220) peaks represent the typical lattice planes observed in MAPbI_3_, which are assigned according to the previously reported XRD pattern in tetragonal phase [S1].

To evaluate the average size of the MAPbI_3_ polycrystalline grains, an optical microscopy measurement was used by a Zeiss Axio Imager 2. The microscope was operated at bright illumination and reflection mode. Using a high-resolution camera combined with the microscopy, **Figure S3 (a)** shows the optical micrographs of the MAPbI_3_ polycrystalline grains, which were densely stacked on a quartz substrate. It shows the same morphology and particle uniformity for the THz time-domain spectroscopy and optical-pump THz-probe spectroscopy in the main text.

In addition, to determine the average particle size, a small portion of the same polycrystalline grains was dispersed on the quartz substrate, as shown in **Figure S3 (b)**. We use the ImagePro software (Media-Cybernetics Inc., Bethesda, MD) to analyze the size of the dispersed MAPbI_3_ grains. The average particle size of the MAPbI_3_ polycrystalline grains is about 10±5 μm.

**Figure S3.** Optical microscopy of MAPbI_3_ polycrystalline grains (a) densely and (b) dispersedly placed on the quartz substrate.

The diameter of one perovskite grain particle embedded in polyethylene (PE) material was measured using scanning near-field optical microscope (nea-SNOM, Neaspec GmbH, Germany), as shown in **Figure S4**. A clear particle size boundary of the perovskite grain is obtained by the second-order demodulation signal (S2). According to the AFM morphology in the near-field characterization, the THz near-field signal exhibits a clear, strong material contrast between MAPbI_3_ grain particle and PE. The particle size can be obtained: x=16 μm, y=8.2 μm, z=2 μm (**Fig. S4 (b)**), which is consistent with the optical microscopic measurement mentioned above.


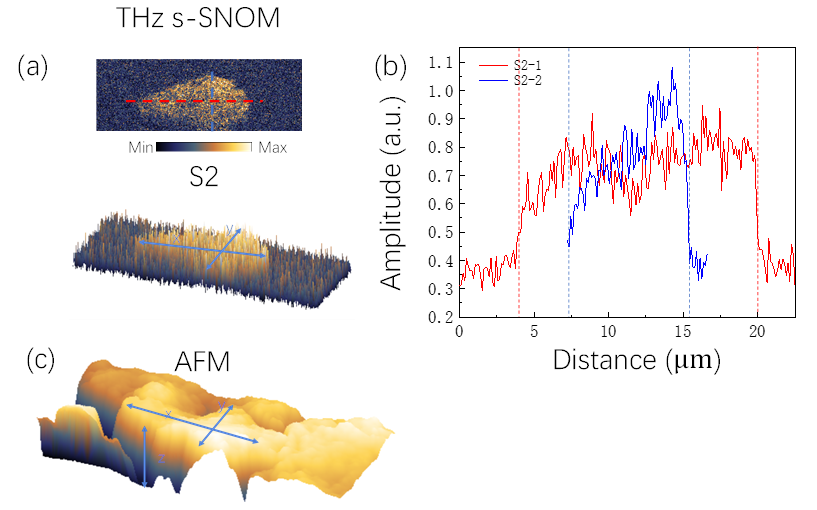


**Figure S4**. (a) THz-s SNOM Optical Near-field characterization of the sample region with a clear boundary between the particles and the PE material. (b) Near-field profile of THz-s SNOM at the blue dashed line and red dashed line in (a), indicating the presence of a clear boundary between the two materials. (c) AFM morphology characterization of the measured particle size, by scanning a perovskite grain particle embedded in PE.

**Note 3. MAPbI_3_** **thin film fabrication** **and characterization.**

The optically smooth MAPbI_3_ thin film was grown to an average thicknesses of 400 nm±100 nm, on 1 cm×1 cm quartz substrate. Firstly, the PbI_2_ film was deposited on the quartz slice by spin-coating a PbI_2_ solution (462 mgml^-1^ in DMF) at 3000 rpm for 30 s. After drying at 70 $℃$, the as prepared PbI_2_ film was dipped in a solution of CH_3_NH_3_I in 2-propanal (10 mgml^-1^) for 20 s and rinsed with 2-propanal, followed by annealing at 100 ^o^C for 1 min in glovebox. **Figure S5** shows the UV-vis absorption spectrum for the MAPbI_3_ thin film.

**Figure S5**. UV-vis absorption spectrum for MAPbI_3_ thin film.

In addition, perovskite Cs_0.05_(MA_0.17_FA_0.83_)_0.95_Pb(I_0.83_Br_0.17_)_3_ thin film was fabricated on 1 cm×1 cm quartz substrate. The preparation of precursor solution and thin film of Cs/MA/FA perovskite follows previous report [S2]. To facilitate the perovskite crystallization, zeolitic imidazolate frameworks were spun on quartz prior to the perovskite film deposition [S3]. The liquid perovskite film was annealed at 100 ℃ for 3 min.

**Note 4. Optical pump/THz probe (OPTP) spectroscopy.**

An OPTP was used to measure the photoconductivity and the carrier mobility of MAPbI_3_. The system is driven by a Ti:sapphire femtosecond amplifier laser operating at a repetition rate of 1 kHz, center wavelength 800 nm, duration 120 fs. The laser pulse was split into three arms: a pump beam, a THz probe beam, and an electro-optic sampling beam, as shown in **Figure S6**. A 400 nm pump pulse was produced by second harmonic generation based on the 800 nm light by beta barium borate (BBO) crystal. The 800 nm or 400 nm pump pulses was used to photoexcite the MAPbI_3_ sample within a ~5 mm diameter area. The transient changes in the THz photo-conductivity were interrogated using a time-delayed THz probe pulse with a$\sim$1 ps-long optical cycle. The THz probe pulse was generated by a 1 mm thick <110> ZnTe optical rectification crystal. The THz probe pulse was focused to approximately 3 mm in size on the sample by parabolic mirrors, ensuring a homogeneous pumping condition. The photogenerated free charge can be accelerated by the oscillating THz electric field, resulting in the attenuation of the THz electric field. Change in the THz transmission through the sample was coherently detected by another 1 mm-thick <110> ZnTe crystal with a Wollaston prism, a pair of balanced photodiodes, and a lock-in amplifier, via a free-space electro-optic sampling beam.

OPTP setup can be operated in two measurement modes. One is a frequency-integrated 1D mode. Keeping the timing of the electro-optic sampling beam fixed to the peak of THz pulse, we scan the timing of the pump pulse relative to the THz pulse. We measure the transmitted THz electric field as a function of pump-probe time delay. The transient dynamics can be probed up to a pump-probe delay of about 400 ps. The time resolution for OPTP measurement is limited by the duration of the pump and sampling pulses. The other is a frequency-resolved 2D mode. We map out the photo-induced THz waveforms $\Delta E_{\mathrm{sample}}$($\omega$,Δt)and obtain $E_{\mathrm{pump}}\left( \omega,\Delta t \right)$ at various pump-probe time delay Δt. Δt is positive when the THz-probe pulse arrives at the sample after the optical excitation. The photoconductivity contains both real and imaginary parts without requiring the use of the Kramers-Kronig relation. To eliminate the absorption of the THz light by water vapor in the air, the system was installed under drying-air condition.

Not that the OPTP experiment is performed in the far field, using freely propagating THz waves. As shown in **Figure S6**, the OPTP experiments probe a macroscopic part of the sample (the cross-section of laser pumping is larger than the THz wavelength). Thus, the sample properties averaged over the probed volume are encoded into the measured signal, involving both the grain boundaries and voids separating the grains. The relevant inhomogeneities are typically much smaller than the THz wavelength.

**Figure S6**. The schematic diagram of our experimental apparatus for the optical pump-THz probe (OPTP) spectroscopy.

**Note 5. Terahertz time-domain spectroscopy (TDS).**

Broadband THz-TDS was carried out on the MAPbI_3_ polycrystalline grains with the EKSPLA system. Femtosecond laser pulses from a 150 mW pump laser of 100 fs duration at 800 nm (repetition rate of 76 MHz) were split into two beams: pump and probe (the split ratio was 50:50). Pump beam modulated by optical chopper was focused on low temperature grown GaAs photoconductive antennas and then emitted THz wave. The diverging THz beam was collected and focused by paraboloidal mirrors to pass through MAPbI_3_ polycrystalline grains. The probe beam through the delay stage was used to detect the THz wave. The effective bandwidth for measured THz signals is from 0.4 to 2.0 THz, the spectral resolution is better than 15 GHz, and the signal to noise ratio (SNR) is larger than 1000:1. All the spectra were performed at room temperature.

In static THz-TDS measurements, we measure the amplitude and phase of the THz pulses transmitted through the substrate alone and the sample on the substrate. **Figure S7 (a)** displays the transmitted time-domain THz waveforms through the empty double-sided adhesive tape, E_Ref._ (t), and through the MAPbI_3_ polycrystalline grains spread on the double-sided adhesive tape, E_Sam._ (t) at room temperature. **Figure S7 (b)** shows the transmitted THz pulses after interaction with the MAPbI_3_ polycrystalline grains, in the absence of a photoexcitation.

**Figure S7.** (a) Measured transmitted time-domain spectra of a blank double-sided tape (Ref., red) and MAPbI_3_ single-crystalline grains spread on the double-sided tape (Sam., blue). (b) THz transmittance over a frequency range from 0.4 to 2.0 THz.

**Figure S8** (a) and (b) show the refractive index and absorption coefficient of the double-sided adhesive tape, which are obtained by inversion of the Fresnel equations, in the frequency range 0.4-1.8 THz, respectively. The mean value of the refractive index is nearly a constant of 1.6 within our THz frequency range. The thickness of the double-sided adhesive tape is around 184 μm.

**Figure S8.** (a) Refractive index dispersion and (b) absorption coefficient of the double-sided adhesive tape at room temperature.

**Note 6. Theoretical calculations of vibrational frequencies.**

We performed the structural optimization and phonon calculations of MAPbI_3_ within the framework of density functional theory (DFT) [S4,S5] using Vienna *ab initio* simulation package (VASP) [S6] based on the projector augmented wave (PAW) method [S7]. The exchange-correlation interaction was treated within the generalized gradient approximation (GGA) [S7] parametrized by Perdew, Burke, and Ernzerhof (PBE). We adopted a $\sqrt{2}\times\sqrt{2}\times2$ MAPbI_3_ unit cell for structural optimization and phonon calculations. The energy cutoff of 500 eV was set in all the calculations and a Monkhorst-Pack grid with $4\times4\times4$ k-points was used for Brillouin zone integration. For the structural optimization, the lattice parameters and all the atoms are fully relaxed until the Hellmann-Feynman forces on all atoms are less than 0.01 eV/Å. The phonon frequencies and eigenvectors for $\Gamma$ point of MAPbI_3_ were calculated using the density functional perturbation theory (DFPT) method. The phonon vibrational displacements of the vibrational modes 24.8 cm^-1^, 49.1 cm^-1^, and 82.9 cm^-1^ are listed in **Fig. S9** and **Table S2**.


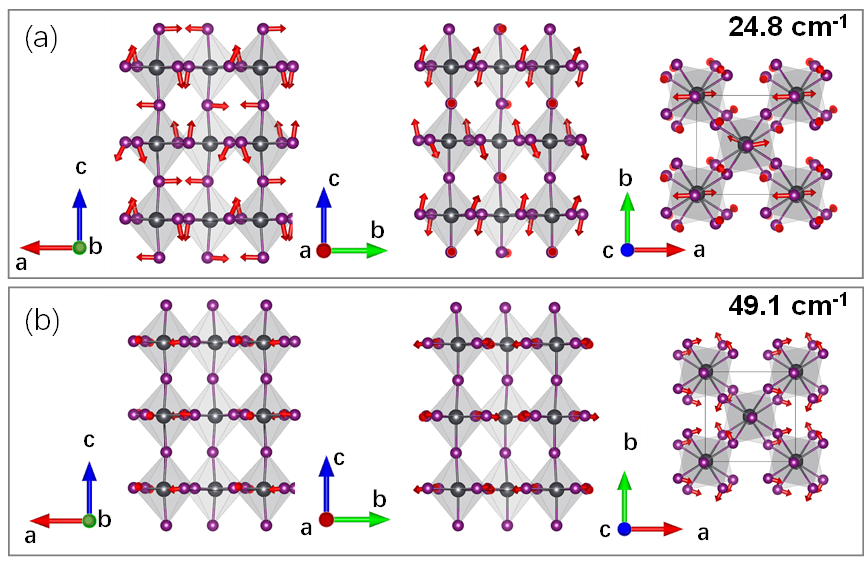


**Figure S9.** DFT calculated skeletal I-Pb-I bending motion of the PbI_6_^4-^ octahedron at (a) 24.8 cm^-1^ and (b) at 49.1 cm^-1^ of MAPbI_3_. Red arrows on the atoms indicate the direction of displacements in the respective vibrational modes. The grey and purple balls denote Pb and I atoms, respectively.

**Table S2.** The atomic positions of I and Pb and vibrational displacements correspond to the vibrational modes 24.8 cm^-1^, 49.1 cm^-1^, and 82.9 cm^-1^.

| Atoms | Atomic positions | | | Displacements | | | | | | | | |
| --- | --- | --- | --- | --- | --- | --- | --- | --- | --- | --- | --- | --- |
|  | x | y | z | 24.8 cm^-1^ | | | 49.1 cm^-1^ | | | 82.9 cm^-1^ | | |
| I1 | 0.07 | 0.09 | 0.10 | -0.18 | -0.23 | -0.11 | -0.07 | -0.03 | -0.01 | -0.02 | 0.06 | -0.18 |
| I2 | 0.07 | 8.81 | 6.66 | -0.18 | 0.21 | -0.11 | -0.07 | 0.03 | 0.00 | 0.02 | 0.06 | 0.18 |
| I3 | 4.40 | 4.36 | 6.66 | 0.17 | 0.22 | -0.08 | -0.07 | -0.03 | 0.00 | 0.01 | -0.05 | -0.19 |
| I4 | 4.40 | 4.54 | 0.10 | 0.19 | -0.22 | -0.09 | -0.07 | 0.03 | 0.01 | 0.00 | -0.06 | 0.19 |
| Pb1 | 1.72 | 6.26 | 0.22 | 0.20 | 0.14 | 0.08 | -0.16 | -0.34 | -0.03 | 0.08 | -0.14 | -0.01 |
| Pb2 | 7.17 | 2.78 | 0.19 | 0.05 | 0.02 | 0.03 | 0.30 | 0.09 | -0.02 | -0.07 | 0.09 | 0.00 |
| Pb3 | 2.74 | 1.81 | 0.22 | -0.21 | 0.15 | 0.13 | -0.16 | 0.34 | 0.03 | 0.09 | 0.14 | 0.00 |
| Pb4 | 6.23 | 7.23 | 0.19 | -0.05 | 0.03 | 0.03 | 0.31 | -0.09 | 0.02 | -0.07 | -0.09 | 0.00 |
| Pb5 | 6.23 | 1.67 | 6.75 | -0.06 | -0.04 | 0.06 | 0.30 | 0.09 | 0.02 | 0.07 | -0.10 | -0.01 |
| Pb6 | 2.74 | 7.09 | 6.78 | -0.19 | -0.14 | 0.12 | -0.16 | -0.33 | 0.03 | -0.09 | 0.14 | 0.00 |
| Pb7 | 7.17 | 6.12 | 6.75 | 0.06 | -0.04 | 0.07 | 0.29 | -0.10 | -0.02 | 0.07 | 0.09 | 0.00 |
| Pb8 | 1.72 | 2.64 | 6.78 | 0.19 | -0.13 | 0.02 | -0.17 | 0.32 | -0.03 | -0.08 | -0.14 | 0.00 |
| Pb9 | 8.78 | 8.81 | 3.42 | -0.24 | 0.13 | -0.03 | -0.05 | 0.02 | 0.04 | 0.02 | 0.00 | -0.37 |
| Pb10 | 8.78 | 0.09 | 9.98 | -0.17 | -0.13 | -0.03 | -0.05 | -0.03 | 0.04 | -0.03 | -0.01 | 0.38 |
| Pb11 | 4.63 | 4.54 | 9.98 | 0.21 | -0.16 | -0.01 | -0.05 | 0.03 | -0.03 | -0.03 | -0.01 | -0.38 |
| Pb12 | 4.63 | 4.36 | 3.42 | 0.22 | 0.17 | -0.01 | -0.05 | -0.02 | -0.04 | 0.03 | -0.01 | 0.38 |

**Note 7. THz photoconductivity spectra of MAPbI_3_ thin film and Cs_0.05_(MA_0.17_FA_0.83_)Pb(I_0.83_Br_0.17_)_3_ thin film.**

**Figure S10.** Real (solid squares) and Imaginary (open points) part of THz photoconductivity spectra in MAPbI_3_ thin film with pump fluence of 30 μJ cm^-2^ (400 nm) at Δt=50, and 100 ps after photoexcitation.

**Figure S10 (a)** and **(b)** show the photoconductivity spectra of MAPbI_3_ thin film measured at Δt =50 ps and 100 ps at room temperature. The flat non-zero value of the real conductivity at low frequency suggests that the photo-induced species in thin film MAPbI_3_ are dominated by the mobile charge carriers. The negative imaginary part is approaching zero at the lowest frequency. The shape of the photoconductivity spectrum changes slightly with increasing time delay. The black solid lines are fits to the Drude-Smith model. The localization parameter c is around $-$0.7, suggesting partial localization of the movement of mobile charge in solution-processed thin film sample. With the pump fluences used here, a mean value of DS relaxation time is around 27 fs for MAPbI_3_ thin film.

**Figure S11.** (a) The time-domain THz signal transmitted through the Cs_0.05_(MA_0.17_FA_0.83_)Pb(I_0.83_Br_0.17_)_3_ thin film. (b) Real part and Imaginary part of THz photoconductivity spectra of Cs_0.05_(MA_0.17_FA_0.83_)Pb(I_0.83_Br_0.17_)_3_ thin film with fluences of 113.2 μJ cm^-2^.

**Figure S11** (a) shows the time-domain THz waveform transmitted through the Cs_0.05_(MA_0.17_FA_0.83_)Pb(I_0.83_Br_0.17_)_3_ thin film before photoexcitation $E_{\mathrm{sample}}$ and pump-induced THz waveform changes $-\Delta E_{\mathrm{sample}}$ of the thin film Cs_0.05_(MA_0.17_FA_0.83_)Pb(I_0.83_Br_0.17_)_3_, at a given pump-probe delay time of $\Delta t=$5 ps. **Figure S11 (b)** show the real part $\sigma_{real,s}^{\mathrm{pump}}$($\omega$) and the imaginary part $\sigma_{imag,s}^{\mathrm{pump}}$ ($\omega$) of $\tilde{\sigma}_{s}^{\mathrm{pump}}\left( \omega,\Delta t=5 ps \right)$ acquired at 400 nm pumping with fluence of 113.2 μJ cm^-2^. Both the ~0.6 and ~1.2 THz resonances are observed.

**Note 8. THz photoconductivity spectra fitted by the DSL model.**

**Figure S12. (a)-(c)** The real and imaginary parts of the sheet photoconductivity spectra of MAPbI_3_ polycrystalline grains. The black curves are the Drude-Smith-Lorentz fits. The dashed and dotted lines show the contributions of Drude-Smith term and Lorentz terms, respectively. **(d)-(f)** The effective scattering rate Γ, plasma frequency ω_p_, and backscattering rate c_1_, at various pump fluences.

**Figure S1****2 (a)-(c)** show the real and imaginary parts of the sheet photoconductivity spectra of MAPbI_3_ polycrystalline grains, measured at ~5 ps after photoexcitation at different pump fluences. The black curves are the Drude-Smith-Lorentz fits. The dashed and dotted lines show the contributions of Drude-Smith term and Lorentz terms, respectively.

Analyzing the complex THz photoconductivity with the DSL model yields effective scattering rate Γ, plasma frequency ω_p_, and backscattering rate c_1_, at various pump fluences, as summarized in **Fig. S12 (d)-(f)**. The mean carrier scattering rate $\Gamma\approx25.8\pm6.0 \mathrm{THz}$ and the factor (1+c_1_)$=0.17\pm0.04$ have been extracted.

**Note 9.** **THz Photoconductivityof MAPbI_3_ polycrystalline grains measured at 5 ps and 200 ps.**

**Figure S13** shows the THz photoconductivityof MAPbI_3_ polycrystalline grains measured at 5 ps and 200 ps. The $\tilde{\sigma}_{s}^{\mathrm{pump}}\left( \omega\right)$ measured at $\Delta t=$200 ps shows a similar spectral characteristic as that measured at $\Delta t=$5 ps after photoexcitation.

**Figure S13.** The real and imaginary components of the THz photoconductivity measured at 5 ps (red) and 200 ps (blue) after photoexcitation for (a) 400 nm, pump fluence of 99 μJ cm^-2^ and (b) 800 nm, pump fluence of 285 μJ cm^-2^.

**References:**

[S1] J. Ding, S. Du, Y. Zhao, X. Zhang, Z. Zuo, H. Cui, X. Zhan, Y. Gu and H. Sun, High-quality inorganic–organic perovskite CH_3_NH_3_PbI_3_ single crystals for photo-detector applications, Journal of Materials Science **52**, 276-284 (2017).

[S2] M.-R. Ahmadian-Yazdi, N. Gholampour and M. Eslamian, Interface Engineering by Employing Zeolitic Imidazolate Framework-8 (ZIF-8) as the Only Scaffold in the Architecture of Perovskite Solar Cells, ACS Applied Energy Materials **3**, 3134-3143 (2020).

[S3] D. Shen, A. Pang, Y. Li, J. Dou and M. Wei, Metal–organic frameworks at interfaces of hybrid perovskite solar cells for enhanced photovoltaic properties. Chemical Communications **54**, 1253-1256 (2018).

[S4] B. Y. Tong and L. J. Sham, Application of a Self-Consistent Scheme Including Exchange and Correlation Effects to Atoms, Phys. Rev. **144**, 1-4 (1966).

[S5] P. Hohenberg and W. Kohn, Inhomogeneous Electron Gas, Phys. Rev. **136,** B864-B871 (1964).

[S6] G. Kresse and J. Furthmüller, Efficient iterative schemes for ab initio total-energy calculations using a plane-wave basis set, Phys. Rev. B **54**, 11169-11186 (1996).

[S7] P. E. Blöchl, Projector augmented-wave method, Phys. Rev. B **50**, 17953-17979 (1994).
